# Experiences of Spanish nurses in the rollout of nurse prescribing: a qualitative study

**DOI:** 10.3389/fpubh.2023.1163492

**Published:** 2023-05-12

**Authors:** Olga Canet-Vélez, Gloria Jodar-Solà, Jaume Martín-Royo, Enric Mateo, Rocio Casañas, Paola Galbany-Estragués

**Affiliations:** ^1^Col·legi Oficial d'Infermeres i Infermers de Barcelona, Barcelona, Spain; ^2^Global Health, Gender and Society Research Group, Facultat de Ciències de la Salut Blanquerna, Universitat Ramon Llull, Barcelona, Spain; ^3^Equipo Atención Primaria Sant Andreu de la Barca, Direcció d'Atenció Primària Metropolitana Sud, Institut Català de la Salut, Sant Andreu de la Barca, Spain; ^4^Unitat Bàsica de Prevenció, Gerència Territorial de Barcelona, Institut Català de la Salut, Barcelona, Spain; ^5^Unitat de Suport a la Recerca Barcelona Ciutat, Fundació Institut Universitari per a la Recerca a L'Atenció Primària de Salut Jordi Gol i Gorina, Barcelona, Spain; ^6^Castelldefels Atenció Primària, Consorci Castelldefels Agents de Salut, Castelldefels, Spain; ^7^Department of Fundamental and Medical-Surgical Nursing, School of Nursing. University of Barcelona, Barcelona, Spain

**Keywords:** nurse prescriber, electronic prescription, nurses, strategies, community care, qualitative

## Abstract

**Introduction:**

Nurse prescribing has legal recognition in Spain, after a long regulatory process, with confusing, changing legislation that does not fully coincide with the reality of nurses' practice. There is currently no research available on how nurses have experienced the rollout of nurse prescribing. The objective of this study is to describe the experiences of nurses in the rollout of nurse prescribing in the province of Barcelona, Spain.

**Method:**

A descriptive qualitative study with intentional sampling was carried out between March 2021 and July 2022. The data were collected through semi-structured individual interviews and discussion groups. The participants were 24 nurses working in the province of Barcelona who were accredited in nurse prescribing or involved in the rollout of nurse prescribing. The data were analyzed using thematic analysis, following Braun and Clark. The COREQ checklist was used to report findings.

**Results:**

We describe nurses' responses on the following themes: internal and external barriers; strategies to support nurse prescribing in the initial rollout and proposals for improvement; and factors linked to nurses' satisfaction.

**Discussion:**

The regulatory process has provided a safety framework for nurse prescribing. Strategies are needed for its comprehensive development and its acceptance among the public. The findings give visibility to nurse prescribing internationally.

## 1. Introduction

Nurse prescribing is currently legal in countries such as the United States, the United Kingdom, Sweden, South Africa, Australia, Canada, New Zealand, Brazil, France, Ireland, Argentina, Norway, Finland, and the Netherlands (1, 2). The application differs across countries, although there are three basic models: independent prescription, supplementary prescription, and prescription based on indications for patient groups (2, 3). In almost all of these countries, nurses are required to receive specialized training before prescribing drugs autonomously (1). In Spain, nurse prescribing for medications and health products has been legal since 2018 (4, 5), after a regulatory process that lasted more than 10 years. As of April 2021, a thousand nurses already had digital cards that allow them to prescribe medications and health products electronically (6).

The concept of nurse prescribing has evolved over the years according to the standards set by national and international bodies. The concept of nurse prescribing arises as a result of this professional growth, as an autonomous, advanced, and specialized practice that is part of the nursing process itself and the nursing care plan (7). Nurse prescribing is defined as the ability to select and indicate techniques, medications, and health products for the benefit and to meet the health needs of the people subject to care during professional nursing practice, under criteria of good clinical practice and clinical judgement (8).

Nurse prescribing has evolved in response to local factors such as the lack of healthcare professionals, the changing needs of patients, and the management of chronic diseases (9). In the last two decades, numerous countries have given nurses the legal authority to prescribe, although legal, practical, organizational, and educational conditions vary considerably from country to country (9, 10).

In studies in Qatar and Australia, researchers found that the development of nurse prescribing contributes to the clinical growth of those who have become prescribers (11, 12). Similarly, in studies conducted in England, researchers have shown that the development of nurse prescribing has allowed nurses to prescribe with confidence (13), improved teamwork (14), and led nurses to be perceived as experts and supportive leaders (15). A study carried out in Ireland found that recently graduated nurse prescribers have high job satisfaction linked to greater autonomy and responsibility (16), and one study in England highlights the importance of nurse prescribers' ability to practice autonomously (17).

The study by Ruiz et al. has shown that the practice of nurse prescribing is associated with better care for people who require healthcare, more effective use of time and resources, and improved cooperation among health professionals (18). Other studies show Irish community nurses' satisfaction with their prescribing role (19) as well as the satisfaction of patients, who positively evaluate their effort in prescribing medications (20). The effects of nurse prescribing on medication and patient outcomes seem positive, although more studies with more rigorous methodological designs are required to draw definitive conclusions (2). Along the same lines, Fox's review (9) emphasizes that the introduction of nurse prescribing must be planned, and the outcomes should be reported, thus supporting its adoption in clinical practice to obtain positive results in the health of the population.

On the other hand, the World Health Organization (21) reaffirms that with limited resources and a tense economic climate, policymakers and health departments must maximize the current potential of healthcare professionals by allowing staff to work within their full scope of practice, optimizing patient and system outcomes. This includes blurring professional boundaries and developing the roles of nursing, pharmacy, and related healthcare professionals to collaboratively meet prescription needs.

At the European level, Irish nurses established nurse prescribing around 2010, and their experience and publications of scientific evidence on the evolution of the prescribing process have made them points of reference in this area. Irish nurses have increased the scope of nursing practice by ensuring safe and effective nurse prescribing (20). As of 2019, 13 countries in Europe have national laws on nurse prescribing. They vary considerably in the medications that nurses can officially prescribe, for what health problems, and under what type of prescription (22).

In the case of Spain, the following nurse prescribing models are used: (a) independent and autonomous nursing prescription (drugs and health products not subject to medical prescription) and (b) collaborative nurse prescribing to an independent prescriber, where sometimes the nurse performs a standardized collaborative prescription and can adjust the dose under established protocols (23). Spanish nurses have extensive training in pharmacology (24), as well as extensive knowledge of health products used by people with chronic health problems, and within their competencies are health promotion and education (18).

A study carried out in Andalusia between 2009 and 2015, showed that the incorporation of nurse prescribing into the public health system meant a significant reduction in healthcare expenditure on health products aimed at the management and monitoring of chronically ill patients, and therefore, an improvement in efficiency (18). Another study carried out in the field of primary care in Catalonia in 2019 showed that nearly 20% of appointments with nurses required at least one prescription and, of these, 72% were prescription drugs and the rest were health products, basically dressings (25). Other studies have shown that nurse prescribing allows the patient to solve health problems with a single professional visit and that, at the same time, contributes to the professional satisfaction, self-esteem, and autonomy of nurses, making it possible to streamline flows and procedures and benefits the multidisciplinary team (26).

In Catalonia, there is consensus on the benefits of nurse prescribing in that it empowers nurses, facilitates the leadership of nurses in care processes, fosters person-centered care, and contributes to the continuity of care (7). To our knowledge, no research describes the process of the rollout of nurse prescribing in Spain from the point of view of the nurses involved, and there is insufficient information on the experiences of prescribing nurses themselves in the international literature. Our qualitative approach uses nurses' opinions and lived experiences, as well as their expertise and knowledge of the context, to understand nurse prescribing from nurses' perspectives.

The main objective of this study is to describe the experiences of nurses regarding the rollout and implementation of nursing prescriptions within Catalonia's electronic prescription system. This objective is divided into three parts: (1) to describe the challenges in the rollout of nurse prescribing; (2) to describe strategies for boosting nurse prescribing; and (3) to describe participants' satisfaction with their role as prescribers.

## 2. Materials and methods

### 2.1. Design

This is a qualitative study with semi-structured individual interviews and discussion groups. We used the consolidated criteria for reporting qualitative research (COREQ) (27).

### 2.2. Setting and participants

The study population was active nurses in the province of Barcelona from two different profiles: (1) accredited nurses who worked in primary care centers, urgent primary care services, and nursing homes in the city or broader province of Barcelona that have pioneered nurse prescribing; (2) nurses involved in the regulation and rollout of nurse prescribing in Catalonia between 2009 and 2019. We used intentional sampling to seek maximum variation in profiles. We excluded nurses who worked in specialized mental healthcare or pediatric centers.

To recruit the sample, we drew on public nurse prescribing data. The Col.legi Oficial d'Infermeres i Infermers de Barcelona (COIB), the province's professional nursing organization, drew up a list of candidates who were accredited in nurse prescribing, currently working, and wrote prescriptions. To select the sample, we sought a balance between homogeneity, which fosters discussion based on shared experience, and heterogeneity, which favors variety. We contacted potential participants by email to inform them of the purpose of the study and verify that they met the inclusion criteria. As for nurses with the profile of experts in the regulatory process for nurse prescribing, they were known figures in the field and therefore we contacted them directly by phone.

### 2.3. Data collection

The two discussion groups were held at the COIB facilities, lasting 60–90 min, and were led by two members of the research group (JM and OC). Individual interviews and discussion groups were audio-recorded, respecting all ethical requirements. The five individual interviews were conducted at the workplaces of each of the nurses interviewed. These interviews lasted 30–50 min and were carried out by a researcher of the project (JM). Table 1 presents the guide used for both discussion groups and individual interviews.

**Table 1:** Guide for discussion groups and individual interviews.

1. How has nurse prescribing evolved in Barcelona? 2. What specific challenges and general challenges have occurred during the rollout of nurse prescribing? 3. What elements can facilitate the process of the electronic prescription led by the prescribing nurse? 4. What barriers must be overcome for nurse prescribing to be consolidated as a health resource? 5. What strategies have been necessary, from the beginning, to make the journey to the present day? 6. What strategies would you propose to support the rollout of nurse prescribing? 7. What should the nursing community do to improve nurse prescribing in the future? 8. What has been your experience as a nurse prescriber? 9. What does being a nurse prescriber mean for your professional career? 10. To what elements or factors would you link your satisfaction?

### 2.4. Ethical considerations

This project was approved by the research ethics committee of the Blanquerna Faculty of Health Sciences, Ramon Llull University *CER-FCSB_10_6_2021*. Participants' confidentiality has been protected according to the European General Data Protection Regulation (RGPD), Regulation (EU) 2016/679, and Organic Law 3/2018 of 5 December on the Protection of Personal Data and Guarantee of Digital Rights. All participants provided written, revocable consent on a paper informed-consent document, before participating in the study. Participants were assigned an alphanumeric code to preserve their confidentiality, and their identities are known only to the principal investigator. Data (recordings, field notes, and transcriptions) are stored in an encrypted external hard drive that is held by the principal investigator.

### 2.5. Data analysis and rigor criteria

The interviews were transcribed verbatim, and a thematic analysis was carried out with the support of the Atlas-ti.9 software. To identify the data, we followed Braun and Clarke's (2014) six phases: data familiarization, initial code generation, theme search, coding, development of themes and subthemes, and writing a final report. Two researchers (OC and RC) independently analyzed the transcripts, carrying out audits. To resolve any discrepancies, the analysis was repeated by a third researcher (PG). Information saturation was reached in the data provided by the participants (28). Credibility was ensured by triangulation of data across participants and cross-verification of the discussion groups, individual interviews, and field notes. To facilitate transferability, we recorded detailed descriptions of participants' demographic information and the data collection methods. To ensure confirmability, we used the audit technique and wrote field notes and memoranda to connect the data and the findings.

## 3. Results

We invited 28 nurses (24 women and four men) to participate in the study. Those who agreed to participate were 24 women: 19 accredited nurse prescribers who participated in the two discussion groups and five nursing experts who had been key figures in the regulation and rollout of nurse prescribing. Because we wanted to draw out the unique expertise of these five participants, we chose to conduct individual interviews with them instead of a discussion group (Table 2).

Table 2Sociodemographic characteristics of participants.

**Table:** 

**Group discussions**							
**Participant ID Code**	**Gender**	**Age**	**Nurse prescribing training**	**Highest level of studies achieved**	**Workplace**	**Worked as a nurse (Years)**	**Worked as a legal NP (Months)**
P1	F	29	–	M	PHC	7	7
P2	F	43	yes	M	PHC	16	3
P3	F	42	yes	M	PHC	22	6
P4	F	32	–	M	PHC	11	10
P5	F	40	yes	M	PHC	20	4
P6	F	39	yes	BN	PHEC	18	3
P7	F	53	yes	SFCN	PHEC	29	3
P8	F	52	–	BN	PHC	27	12
P9	F	31	–	BN	PHC	5	12
P10	F	53	–	DS	PHC	31	6
P11	F	43	yes	BN	PHC	25	5
P12	F	37	yes	M	NH	13	12
P13	F	45	yes	M	NH	28	12
P14	F	44	yes	SFCN	PHC	20	6
P15	F	31	–	M	PHC	7	1
P16	F	43	yes	M	PHC	27	4
P17	F	30	yes	M	PHC	8	6
P18	F	35	–	SFCN	PHC	11	5
P19	F	30	–	BN	PHC	7	6

**Table:** 

		**Individual interviews**			
**Participant ID Code**	**Gender**	**Age**	**Years in management**	**Highest level of studies achieved**	**Professional role function**
P20	F	58	30	PhD	Ma, NT, SFCN
P21	F	70	35	BN	Ma, NT
P22	F	51	10	PhD	Ma, NT
P23	F	54	25	DS	Ma, NT, SFCN
P24	F	45	20	SFCN	Ma, NT, SFCN

BN, bachelor of nursing; DS, doctoral student; F, female; M, master's; Ma, manager; NP, nurse prescriber; NT, nursing trainer; PhD, Doctor; PHC, primary health center; PHEC, primary health emergency center; SFCN, specialist in family and community care nursing.

The nurses who participated in the discussion groups had an average age of 41.1 years and professional experience of 17.5 years (between 5 and 31 years), and 58% had completed nurse prescribing training. Of this group, 63% had a master's degree and 17 of the 19 nurses worked in primary care centers. The expert nurses who participated in the individual interviews had an average age of 55 years, 24 years in professional practice (between 10 and 35 years), and all held administrative, management, and training positions.

The results are described below according to the three study objectives.

### 3.1. Challenges of the rollout of nurse prescribing (Objective 1)

Table 3 contains the themes and subthemes that we detected in our analysis for objective 1. It describes both internal and external barriers.

**Table 3:** Themes and subthemes related to the challenges of nurse prescribing.

**Objective 1**	**Themes**		**Subthemes**
Challenges in the rollout of nurse prescribing	Barriers to development	Internal	- Constant changes and innovation in nursing care - Unequal professional self-recognition - Lack of training in pharmacology
		External	- Very restrictive catalog, and a physician has to sign off on prescriptions by nurses - Nurse prescribing was not regulated outside the treatment plan - Complex administrative procedure - Lack of confidence in nursing skills

#### 3.1.1. Barriers to the rollout of nurse prescribing

We detected two types of barriers in the regulation of nurse prescribing: internal barriers created from within the nursing community and external barriers.

##### 3.1.1.1. Barriers internal to the nursing community

Participants reported that nursing care is in constant change to respond to the needs of the population. This is stressful and often divides a very plural group, who do not always believe in the value of nurse prescribing.

“*The great barrier is that we still don't fully believe in the importance of nurse prescribing.”* ***P24***

“*We never stop evolving and it's non-stop... super stressful. Because of this we end up being a divided group, because of so many changes. And in the end, nurse prescribing is just one more thing.”* ***P6***

Some nurses do not see nurse prescribing as indispensable or do not see themselves as prescribers for reasons such as a lack of training in pharmacology or a lack of conviction in their capacity to develop these skills.

“*I think we're still very divided between nurses not seeing themselves as prescribers, not feeling like prescribers. And nurses who do. Sometimes we have to look at ourselves, inside the profession, where we have strengths, where we have weaknesses, and here I think it's a weakness: where we don't all stick together”* ***P7***

“*I believe that from the school of nursing or from the health center, they need to make a move and truly train people who have more difficulty prescribing.”* ***P6***

##### 3.1.1.2. Barriers external to the nursing community

Having a legal framework to prescribe gave recognition to nurse prescribing as a function that is integrated into the patient record that is shared among professionals. Achieving a regulatory framework was the first challenge, and it required effort and perseverance. Still, the participants described the catalog of medicines and devices that nurses can prescribe as very restrictive and incomplete. In addition, in some areas or situations, such as in emergencies, home care, or care for chronic patients, the nurse's word is not final (a physician must sign off on the prescription).

“*In primary and community care, they are super in favor of nursing broadening its catalog of pharmacological prescription, because they rely on the training and preparation of nurses. In the hospital environment, there's a certain fixation with the idea that nurses can't prescribe”* ***P6***

“*We in the emergency room, in addition to the fact that we have acute demand, there are very clear protocols, which is like if you diagnose a streptococcal infection, you follow the protocol, you have a case history and you have to prescribe an antibiotic. But you have to go to the doctor to sign it for you.”* ***P5***

It would be an advance if the prescription was regulated and collaborative into the treatment plan of the patient. Nurse prescribing is not limited to medications and devices; it also applies to diet, physical activity, and social issues, aspects that are inherent to nursing.

“*Therefore, here we need a lot of progress toward collaborative prescribing based on protocols and clinical practice guidelines.”* ***P20***

“*Nurse prescribing is the nursing activity that points out the best treatment regimen, but it doesn't necessarily have to include a drug.”* ***P21***

The administrative process for obtaining the card that makes it possible for nurses to prescribe is complex and lengthy. Currently, not all nurses have done so yet. Nurses must take the initiative to request the card and then the health center must certify their length of employment.

“*We have a lot of problems with people who didn't start the process at the time... and that today still don't have the card for prescribing, and that generates a lot of frustration.”* **P4**

“*The whole bureaucratic procedure has been a bit complicated... through the company how to request it. They should make it really easy, so these nurses could call a single phone number and find out how to get the card and make it really easy.”* ***P24***

The participants detected a lack of confidence in their skills and responsibilities among some health professionals and part of the population, reflecting misinformation about nurse prescribing.

“*To this day, the vast majority of the population does not know that nurses can prescribe and neither do nurses themselves understand very well what they can prescribe*.” ***P24***

“*I believe that when we are aware of the responsibility that the nurse has when we go to work on the treatment plan jointly, the added value of nurse prescribing […] will be appreciated”* ***P23***

### 3.2. Strategies for boosting nurse prescribing (Objective 2)

In this section, we describe past and present actions taken to boost nurse prescribing and also the participants' ideas for how to improve in future (Table 4).

**Table 4:** Themes and subthemes related to strategies for boosting nurse prescribing.

**Objective 2**	**Themes**	**Subthemes**
Strategies for boosting nurse prescribing	Actions taken during the initial rollout	- Leadership and active participation of nurses - Explaining nurses' preparation for prescribing - Demonstrating the scientific evidence surrounding the benefits of nurse prescribing from other countries
	Areas for improvement	- Increase the catalog of drugs, supplies, and treatments that nurses can prescribe - Normalize nurse prescribing among the public - Promote nurse prescribing within the nursing community itself, with respect to other consolidated roles - Provide recognition and external support for the responsibility inherent in nurse prescribing - Provide continuous training in pharmacology - Change the “medication plan” to a “treatment plan”

#### 3.2.1. Actions taken during the initial rollout

Participants pointed out the leadership and active participation of the COIB at the beginning of the regulatory process for nurse prescribing, and later its perseverance in ensuring that nurse prescribing would be integrated into the electronic prescription system. They also recall the support of the Catalan Ministry of Health at important times, such as the approval of Royal Decree 180/2019.

“*At the time, remember who was the Minister [of Health], which is the one who signed so that [nurse prescribing] could be developed here in Catalonia... Then the Col.legi [COIB], [worked on] [...] professional development along with key elements for working on this topic”* ***P24***

It was necessary to demonstrate the nurses' competence in prescribing. The curricula of the nursing degree had to be reviewed to document nurses' training in clinical pharmacology, which had always been present in the curriculum. It was also demonstrated that nurse prescribing was extensive in other countries, and there was scientific evidence about its benefits in different specialties.

“*Having a nursing degree already accredited you sufficiently for the training received... in addition, novice nurses are accredited through an extra course in prescription“**P21***

“*... Nursing must ensure its competence. Our role in prescribing is endorsed in publications with results showing good practices and benefits…. We just need to be allowed to perform [prescriptions] and not be questioned.”* ***P5***

#### 3.2.2. Areas for improvement

Participants stressed the limits of the current prescribing catalog for nurses.

“*The catalog is not real in terms of the activities that we carry out. It's necessary to expand the products and drugs. If they don't increase, nurse prescribing might not [cover] the things that are necessary for the patient, although it includes a series of products that are obviously important in our daily work, but insufficient.”* ***P23***

“*We need the Col.legi [COIB] to work to expand this catalog.”* ***P19***

Participants reported that it was essential to normalize nurse prescribing among the public as well as empower nurses to prescribe. This would mean carrying out the complete care process, from the detection of needs to the action to solve the detected problem. This would contribute to a more effective service covering patients' needs, thanks to the development of nursing competencies.

“*We will only finish consolidating nurse prescribing when citizens normalize it, so that they see it as good for them, the results in terms of people's health.”* ***P21***

“*As a nurse, I have prescribed medications; I have changed doses. And in an ICU [intensive care unit] also you might incorporate medications, you modify some and you remove others. So the issue is to normalize this.”* ***P15***

It would be positive to carry out actions in the media to make nurse prescribing known to the public, as well as to provide scientific evidence about its usefulness and benefits.

“*If nurses are really so important, we'll give them the credit they deserve, and in this case, because if prescribing is very important, well you have to inform the public.”* ***P24***

Participants described having to defend themselves as prescribers, which generates insecurity and burnout.

“*It's a cultural issue. We're still a female profession, with all that it brings. We have to justify what we do, why we do it. Also because we're constantly changing.”* ***P2***

“*We have gone to pharmacy sessions and they say to us, ‘But why are you here?'. Well, because we also prescribe. In nurse prescribing everyone has to stay in line. If the doctor says ‘I'll take this [medication] away [from the patient]', nurses can comment from an autonomous point of view or a collaborative one.”* ***P4***

“*There's been a change in the hospital, it [nurse prescribing] has been valued more, but the doctors have their speciality and we're more general. The doctor has a well-defined place, and we have to justify ours. This wears us down a lot, explaining why nurse prescribing needs to exist.”* ***P4***

Disciplinary inequalities must be resolved. For example, participants wondered why resident nurses in a specialty cannot prescribe when medical residents can.

“*But specialist nurses in the second year cannot prescribed, but medical residents can.... This has been reported to management.”* ***P8***

“*Promote the culture of prescribing so that really when undergraduate nursing students come to do their practicum, they have to internalize the issue of nurse prescribing, just like when doctors finish they have it [internalized].”* ***P3***

Nurses' predisposition toward prescribing would improve if the responsibility it implies were recognized and supported. Not all nurses want to prescribe, but this should not be an individual choice. In other types of nursing activities, optionality would be unthinkable. For this reason, it is necessary to focus on the causes of this reluctance, be it the will, the knowledge, or the attitudes of nurses.

“*Many think, they feel that this increase in responsibility is not supported by anything, that it's not recognized.”* ***P8***

“*Many think, they feel that this increase in responsibility is not supported by anything, that it's not recognized”* ***P8***

“*But that's it, I believe that the issue of compulsory education is precisely one of the problems we have, because if you always have the possibility of not doing it, you can think, ‘Tomorrow someone else will come and they'll do it'. Can you prescribe or not? If not, get training.”* ***P13***

“*We are trained, we have training, and those who don't [having training] need to get trained. Because the problem of a lot of nurses sincerely is assuming that responsibility. ‘Do I want to assume that responsibility or not?”'* ***P6***

Achieving a change from a “medication plan” to “treatment plan” would be an improvement. This modification would involve incorporating pharmacological treatment into a more global and integrative perspective, which would contribute to the quality of care.

“*To look for points where the two groups [nurses and physicians] can come together and complement each other. […]”* ***P11***

### 3.3. Participants' satisfaction with their role as prescribers (Objective 3)

This third objective was to explore participants' satisfaction with nurse prescribing and the impact of being a nurse prescriber on their professional careers (Table 5).

**Table 5:** Themes and subthemes on the satisfaction derived from nurse prescribing.

**Objective 3**	**Theme**	**Subthemes**
Participants' satisfaction with their role as prescribers	Positive aspects	- Feeling empowered - Being recognized for their role and competencies - Benefits for patients and the general public - Aspects related to colleagues on the team - Milestone achieved by the nursing community

#### 3.3.1. Sources of satisfaction

Participants reported positive experiences with nurse prescribing and linked this satisfaction to several factors. Participants perceived nurse prescribing as a process by which nurses increase their decision-making and leadership capacity. This led participants to feel more empowered in their workplace, as their roles and competencies are recognized, and they become reference points both for patients and colleagues on their team. Nurses who have pioneered nurse prescribing have become role models for nurses who are gradually taking on this role.

“*Satisfaction [for me] is the recognition for the work that I, that nurses, have been carrying out for a long time. And the benefit it brings to the population of not having to wait for a prescription to be validated [by a physician].”* ***P4***

This is recognition of a profession that has opted to expand its functions in a regulated way. After a long wait, the fact that the nurse's signature is finally present on electronic prescriptions just like other professionals is seen as a milestone for nursing.

“*I'm celebrating because it was something that we were waiting for.... It has been possible to make this card a reality, when it seemed it would never happen.”* ***P24***

“*I thought the moment would never arrive when I could end my visit with a person by prescribing care that is included in the medication plan. For me it has been comforting.”* ***P20***

## 4. Discussion

### 4.1. Summary of evidence

Our analysis sheds light on nurses' initial experiences of the rollout of nurse prescribing, which can serve as a reference to other countries that are undertaking this process. The prescription of medications by nurses is increasing worldwide, but research in Europe is limited (22). No review of the rollout of nurse prescribing exists (9), and there is no previous data on nurses in our context, especially from a qualitative perspective.

The results of the study also point to the limited visibility of nurses in clinical practice, possibly due to a lack of indicators, as highlighted by Nascimento (29). Another finding relates to the complex phenomenon of professional responsibility in the practice of nursing (30) and the empowerment of nurses through specific competencies such as nurse prescribing. True empowerment involves a nurse controlling her practice (31), and at this early stage of the rollout of nurse prescribing in Barcelona, this has not yet occurred.

The participants reported that the regulation of nurse prescribing in Barcelona has provided a legal framework as well as the recognition of their authority and autonomy. Nurse prescribing is extensive in other countries and has clear benefits, as seen in studies that show a higher demand for incontinence prescriptions directed by nurses (32).

Our participants' satisfaction with nurse prescribing is high, coinciding with patient satisfaction found in a study by Duarte (33). Nurses achieve higher levels of patient satisfaction, compared to primary care physicians (34, 35).

At the same time, we learned that not all nurses want to prescribe. Perceptions of competence, role, and risk influence the decision to prescribe (36), but this tendency would likely change if the responsibility assumed by nurse prescribers were recognized and supported by health managers, professional teams, patients, and the general public (37). Nuttall's (3) study reveals that some primary care nurses did not see nurse prescribing as part of their role, but overall nurse prescribing was seen as an essential component of nursing, especially for specialist nurses. Numerous barriers remain for the full implementation of nurse prescribing, and overcoming them requires a more coordinated approach (38). Other studies show that the lack of recognition of nurse prescribing is coupled with the lack of knowledge on the part of the population about the rigorousness of the nursing field (39). Ignorance of the regulations and conditions governing the prescription of medications is identified as a universal problem, which is why it is important to inform colleagues and multidisciplinary teams (19). Nurses seek the competency of prescribing, for their professional development and the efficiency of the public health system (40, 41), in line with health managers, who are interested in authorizing and expanding this competency, considering how it benefits patient safety and the health system itself (18).

Fox's review (9) offers practical recommendations for the implementation and adoption of non-medical prescriptions by the different agents involved. Implementation requires significant organizational support and careful planning to maintain interdisciplinary relationships and clarify roles and responsibilities. Also, to allow optimal uptake of nurse prescribing, it is necessary to earn the trust and support of patients, clinicians, and health administrators. The legalization of nurse prescribing also implies changes in power and inter-professional relationships, requiring adjustments to enable the effective implementation of nurse prescribing (3).

Participants identified the need for continuous training in clinical pharmacology, at the same time deprescribing must also be considered, from the non-medicalised perspective of the population. For this reason, the analysis of the types of nurse prescribers, prescription restrictions, and the necessary supervision models is key (42). There is an ongoing need to help nurses increase their knowledge and skills to support their prescribing role (43).

Hindi et al. (44) show that there are still barriers to the implementation of independent nurse prescribing in primary care teams. They argue that for nurse prescribing to be efficacious, there must be appropriate training for nurse prescribers, effective integration with the rest of the primary care team, and acceptance by patients. Graham-Clarke et al. (45) show that nurse prescribing seems to be easier to adopt when it is part of the person's overall care. When new roles need to be established, nurse prescribing takes longer to be universally adopted.

Nurse prescribers can improve people-centered care (3). It makes it possible to provide holistic health promotion (46), and nurses prescribe for a wide range of patients in a manner comparable to that of physicians (2).

We need more studies about the effects of nurse prescribing, and for it to thrive, it must be recognized, understood, and included in organizational processes. At the same time, we must address the challenges that nurse prescribers encounter during their daily practice. Moreover, on the other hand, university schools of nursing need to promote nurse prescribing.

### 4.2. Limitations of this study

Qualitative studies with small samples are useful for identifying questions that deserve further research, but they have a limited capacity for generalization. The sample size was adequate for our purposes because data saturation was reached on the topic of the participants' experiences surrounding nurse prescribing. However, these results cannot necessarily be extrapolated to other healthcare systems, so care must be taken in drawing comparisons internationally. Nonetheless, they may be relevant in other countries where nurse prescribing is in the regulatory phase or its initial rollout. In addition, this was a short-term study, while prolonged engagement with participants can provide a valuable way to identify present and future challenges. By including only the nurses who had the most experience with nurse prescribing, we may have missed the reasons why nurses at other centers are not prescribing as much. Future studies should consider a broader range of accredited nurse prescribers to give us a fuller picture. Finally, in retrospect, the wording of our question about satisfaction with nurse prescribing could have conditioned the responses. However, the impact on the overall data was minimal because this was the last question we asked. Moreover, when asked this question, several nurses told us about challenges and barriers (rather than satisfaction), suggesting that the question did not prevent negative opinions from surfacing.

### 4.3. Implications for clinical practice

This study may be useful for health administrators and policymakers. The findings of this study are relevant for clinical practice internationally, as they provide suggestions for how to facilitate the rollout of nurse prescribing, increasing the efficiency of public services, especially given the current shortage of health professionals worldwide. Health centers can call on nurse prescribers as they redistribute care activities to make the best use of available resources. Nurse prescribing could be crucial in improving the efficiency and quality of treatment plans. Also, understanding nurses' experiences can help professional associations and schools of nursing tailor their training in nurse prescribing and support nurse prescribers.

## 5. Conclusion

The regulatory process for nurse prescribing has lasted a long time but has provided a legal and safety framework. We detected barriers to nurse prescribing both inside and outside the nursing community that must be overcome in order for nurse prescribing to be consolidated within clinical practice. At the same time, we identified present and future strategies to facilitate the development of nurse prescribing, which will affect the nurses themselves, the population cared for, and the management of public health services.

## Data availability statement

The raw data supporting the conclusions of this article will be made available by the authors, without undue reservation.

## Ethics statement

The studies involving human participants were reviewed and approved by Research Ethics Committee of the Blanquerna Faculty of Health Sciences, Ramon Llull University CER-FCSB_10_6_2021. The patients/participants provided their written informed consent to participate in this study.

## Author contributions

PG-E, GJ-S, and OC-V contributed to funding acquisition and design of the study. JM-R and OC-V performed the data collection. OC-V and RC performed the analysis and interpretation of data. OC-V and RC wrote the first draft of the manuscript. PG-E, EM, GJ-S, and JM-R involved in drafting the manuscript or revising it critically for important intellectual content. All authors read and approved the final version of the manuscript.
